# Self-powered thin-film motion vector sensor

**DOI:** 10.1038/ncomms9031

**Published:** 2015-08-14

**Authors:** Qingshen Jing, Yannan Xie, Guang Zhu, Ray P. S. Han, Zhong Lin Wang

**Affiliations:** 1 Department of Materials Science and Engineering, College of Engineering, Peking University, Beijing 100871, China; 2 School of Materials Science and Engineering, Georgia Institute of Technology, Atlanta, Georgia 30332, USA; 3 Beijing Institute of Nanoenergy and Nanosystems, Chinese Academy of Sciences, Beijing 100083, China

## Abstract

Harnessing random micromeso-scale ambient energy is not only clean and sustainable, but it also enables self-powered sensors and devices to be realized. Here we report a robust and self-powered kinematic vector sensor fabricated using highly pliable organic films that can be bent to spread over curved and uneven surfaces. The device derives its operational energy from a close-proximity triboelectrification of two surfaces: a polytetrafluoroethylene film coated with a two-column array of copper electrodes that constitutes the mover and a polyimide film with the top and bottom surfaces coated with a two-column aligned array of copper electrodes that comprises the stator. During relative reciprocations, the electrodes in the mover generate electric signals of ±5 V to attain a peak power density of ≥65 mW m^−2^ at a speed of 0.3 ms^−1^. From our 86,000 sliding motion tests of kinematic measurements, the sensor exhibits excellent stability, repeatability and strong signal durability.

## Abstract (web summary)

Kinematic sensors are required in many industrial applications, but the current sensor designs rely on power input from external sources. Here, Jing *et al.* harness the micro-meso scale ambient energy via a triboelectric generator to self-power sensors for one- and two-dimensional motion vector sensing.

## Graphical abstract

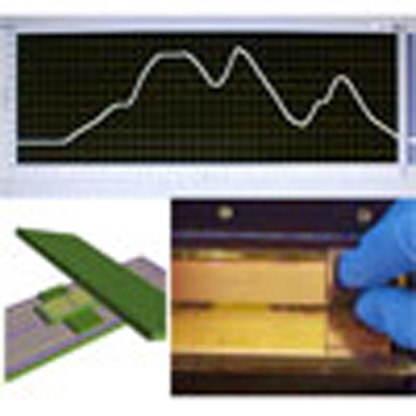

Kinematic sensing for displacement and velocity vectors is in high demand in manufacturing, transportation, robotics, mapping and so on. Current technologies for these vector measurements are based on capacitance^1^,^2^,^3^,^4^, electromagnetic interaction^5^, optical sensing^6^ and so on; however, they all require an external power source to operate. Rapid advancement in the development of a triboelectric nanogenerator (TENG)^7^,^8^, which includes experimental^9^,^10^,^11^ and theoretical models^12^ has generated a whole new class of self-powered sensors: chemical sensors^13^,^14^, velocity sensors^15^,^16^,^17^, magnetic field sensors^18^, pressure sensors^19^,^20^,^21^, acoustic sensors^22^, touch sensors^23^,^24^, position sensors^25^,^26^ and so on. For the velocity sensor, which is of interest to this paper, the reported design^15^ utilizes electrodes of varying lengths to detect motion direction by comparing the sequence of pulses with different amplitudes. These characteristics, however, could not be reliably employed for high-speed direction sensing, which requires continuity, fast response and high sensitivity.

In this work, a self-powered thin-film kinematic vector sensor for measuring displacement, velocity, acceleration and their directions is presented. By embedding two-column arrays of copper electrodes into the polytetrafluoroethylene (PTFE) film of the mover and polyimide (Kapton) film of the stator, triboelectric signals are generated during the relative reciprocation of the mover to the stator. To sense its moving direction, two sets of electrodes containing a small phase difference are used as markers and built into a direction-sensing TENG (dsTENG) unit for the one-dimensional (1D) and two-dimensional (2D) motion measurements. Errors of less than 1% in multiple velocity measurements are achieved and accelerations from 0.1 to 0.5 m s^−2^ are continuously tracked by the sensor. The self-powered device is not only relatively simple to fabricate and install, it is able to generate robust signals that are highly repeatable, and, therefore, we believe that our demonstration of the applied triboelectric technology will greatly impact the industrial sensor-manufacturing sector.

## Results

### Structural configuration

**Figure 1:**
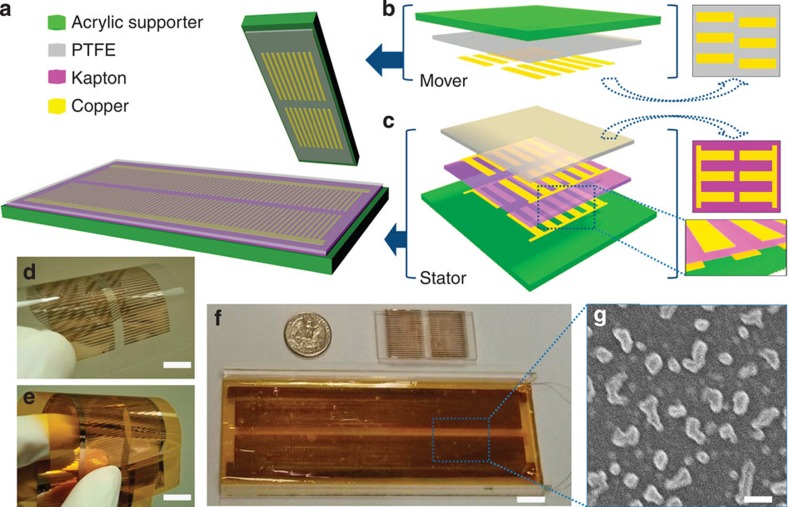
Schematic and experimental structure of a dsTENG. (**a**) Schematic structure of a dsTENG. (**b**) Detailed composition layers for the mover part and (**c**) for the stator part. (**d**) Experimental fabrication of the PTFE film for the mover (scale bar, 1 cm) and (**e**) of the Kapton film for the stator (scale bar, 1 cm). (**f**) Assembled experimental demo (scale bar, 1 cm). (**g**) Teflon nanoparticles spread on the PTFE film (scale bar, 1 μm).

The dsTENG (Fig. 1a) consists of a mover (Fig. 1b) and a stator (Fig. 1c) that are fabricated from several layers of highly pliable thin films. The mover consists of a PTFE thin film deposited with two columns of 500-μm-wide copper electrodes with a linear pitch of 1 mm (Fig. 1d) on the surface that contacts with the stator. The electrodes of one column are placed at a quarter pitch offset with respect to the other column (Fig. 1b and Supplementary Fig. 1). The stator consists of a Kapton film^27^ with both surfaces deposited with two fully aligned columns of copper electrodes (Fig. 1c,e). The electrodes are placed on the top and bottom sides of the Kapton film at a linear shift of half of a pitch, which results in an alternating electrode pattern with one another (Fig. 1c and Supplementary Fig. 1). All the copper electrodes are identical in dimension and when a relative movement occurs between the mover and stator, the electrode arrangement between the two contacting surfaces generates an alternating pattern of the electrodes coming into repeated alignments and misalignments. To tap the resulting triboelectrification energy, the electrodes from one column of the Kapton film are connected via bus electrodes to form a top electrode (TE) on the upper surface of the Kapton film, and a bottom electrode (BE) for the ones on the bottom surface of the Kapton film. This two-side placement of electrodes is in contrast to the one-side placement of electrodes^28^; a high-resolution motion sensing is almost impossible with the latter design. Together, the TE and BE form an electrode pair marked as a base channel (BC), with the other pair assigned as a reference channel (RC). The TE and BE from one channel are electrically connected via an external load for signal measurements (Supplementary Fig. 2). A thin PTFE film covers the Kapton layer, and it acts not only as a protector of the electrode channels but also as a high-performance electrification material for generating the triboelectric charges during the relative sliding motion. All the films used are thin and highly pliable and, thus, can be easily bent to fit a curved surface (Fig. 1d,e). For our demonstration, the films are attached to a flat rigid acrylic holder (Fig. 1f). To mitigate frictional effects during relative mover–stator motions, a Teflon nanoparticle spread is applied to the PTFE film (Fig. 1g) to act as a lubricant and our numerous testing showed that the nanoparticles do not appear to adversely affect the triboelectrification process^27^,^29^.

### Signal generation process

**Figure 2:**
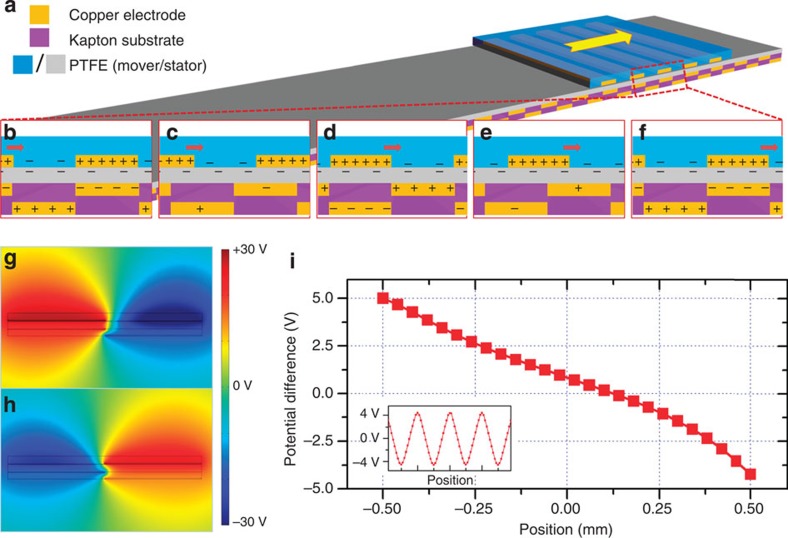
Working mechanism and simulation results. (**a**) Charge transfer mechanism of the dsTENG. (**b**–**f**) Local charge behaviour when mover is at different positions during sliding. (**g**) Potential simulation when copper strips on the mover are aligned with the TE strips. (**h**) Potential simulation when copper strips on the mover are aligned with the BE strips. (**i**) Output voltage over positions between the two situation shown in **g**,**h**.

The mechanism for generating the triboelectric potential of a single channel is illustrated in Fig. 2a. Owing to the coupling of the triboelectrification and electrostatic effects when the PTFE and copper are brought into relative motion (Fig. 2a), electrons will be periodically driven to flow forward and backward between the TE and BE through an external circuit. Figure 2b–f depicts the charge response in a single channel. As the mover slides over the stator, electrons from copper strips are injected into the PTFE film by the triboelectricity^28^. Owing to the differential contact areas and to maintain a zero total charge balance, the local charge density in the copper is higher than that in the PTFE. When motion occurs, charges in the strip pattern generates a corresponding electric potential distribution in the surrounding space that periodically attracts or excludes electrons from the neighbourhood electrodes, resulting in an a.c. output. Hence, the dsTENG begins producing kinematic-sensing signals as soon as relative motion occurs between the contact interfaces of the mover and stator. For the dsTENG to sense direction vectors, we employed an assaying technique to keep track of the signal cycles against the phase difference between the BC and RC.

The following electrostatic simulation can be used to better understand the process. When copper strips on the mover are aligned with the TE strips (Fig. 2g), the TE is associated with the positive potential, which implies that the BE is negative. As they become aligned with the BE, the potential reverses sign (Fig. 2h) and the open-circuit voltage (*V*_oc_) between the two electrodes can be computed from the following equation using Gauss theory. Details are presented in Supplementary Fig. 3 and Supplementary Note 1:


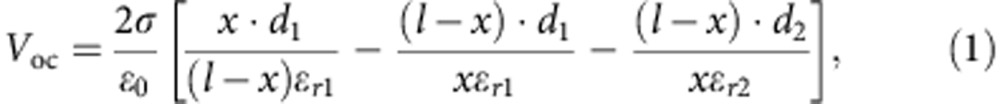


where *σ* is the triboelectric charge density on PTFE; *ɛ*_0_ is the permittivity constant in vacuum; *d*_1_, *ɛ*_*r*1_, *d*_2_, *ɛ*_*r*2_ are the thickness and relative permittivity of the PTFE film and Kapton film on the stator, respectively; *l* is the width of the electrode and *x* is the relative displacement between the mover and the stator. Figure 2i shows a plot of *V*_oc_ against position. Owing to the non-symmetry of the structure, the output is also non-symmetric; however, this will not affect the sinusoidal output sketched in the inset of Fig. 2i.

When the TE and BE are connected by an external load *R*, electrons begin to flow between these two electrodes. The response is governed by a pair of differential equations (Supplementary Note 1):


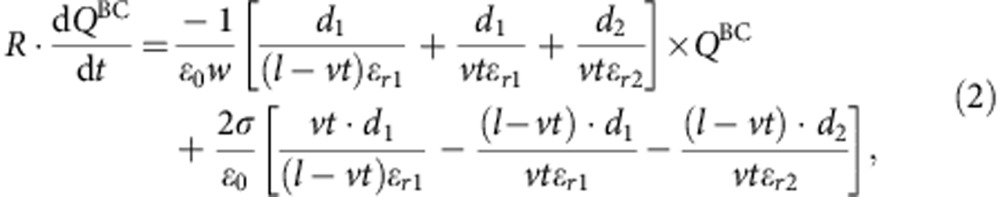



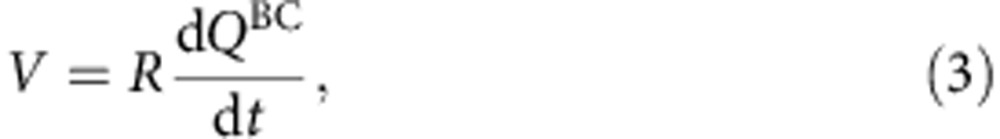


in which *Q*^BC^ is the charge transfer from TE to BE and *v* is the relative velocity.

### Optimization

**Figure 3:**
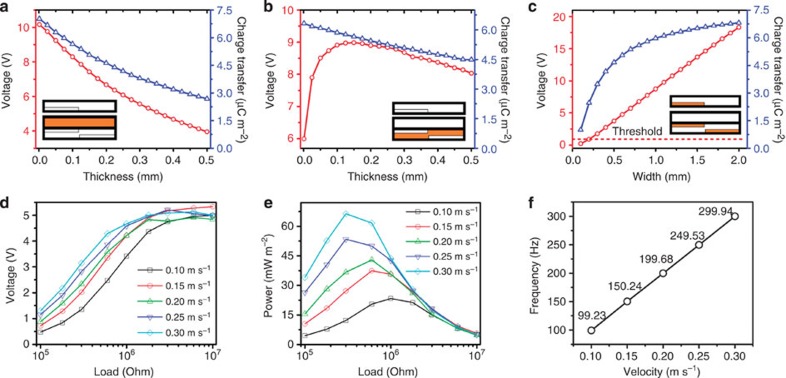
Characteristic studies. Theoretical simulation results on the (**a**) effect of the thickness of PTFE film on stator; (**b**) effect of the thickness of Kapton film and (**c**) effect of the width of the copper electrode. Insets in (**a**–**c**) indicates the variable reflected on the structure. Experimental measured results for a fabricated dsTENG. (**d**) Output voltage over different external resistance. (**e**) Output power over different external resistance. (**f**) Dominant output frequency (abstracted from fast Fourier transform (FFT)) over different moving velocities.

The output signal is affected by factors such as the thickness of PTFE and Kapton films, the width of the copper strips, external load and sliding velocities. To determine the optimum magnitudes, we numerically simulate both the output voltage *V*_oc_ and the total charge transfer (*Q*_sc_) via equations (1)–(3), 2, 3 by varying the thickness of the PTFE and Kapton films and the width of the copper electrodes (Fig. 3a–c). As the thickness of the PTFE film (in stator) increases from 1 to 500 μm (Fig. 3a), the output voltage decreases 61% from 10.0 V at 1 μm thickness and the average charge transfer density decreases 62% from 7.0 μC m^−2^ at 1 μm thickness. This is because a PTFE behaves similar to a dielectric layer, and the thicker it is, the more it reduces the penetration of the electric field in the mover. Thus, for maximum output voltage and charge transfer, the protective PTFE layer should be as thin as possible. Similarly, when the thickness of the Kapton film is increased from 1 to 500 μm (Fig. 3b), the output voltage first increases rapidly from 5.9 V at 0 thickness (for electrodes placed on the same side) to a peak value of 8.9 V at 125 μm and then decreases gradually to 8.0 V at 500 μm as the adverse capacitive effect between the electrodes kicks in. Likewise, the average charge transfer density decreases 29% from 6.3 μC m^−2^ at 1 μm thickness as the film thickness is increased. The simulation results suggest that the Kapton film should have an optimized thickness of between 75 and 150 μm to generate the highest voltage outputs. Another useful result of the simulation is that it clearly shows the advantage of placing the electrodes on two sides of the Kapton film versus placing them all on one side as in the design of Leng *et al.*^28^. The output voltage of 8.9 V for the two-side placement is 50% higher than the one-side placement of electrodes. Next, we consider a parametric study of increasing the width of the copper electrodes from 100 to 2,000 μm (Fig. 3c). It generates a linear increase in the output voltage and a decelerating exponential growth in the charge transfer. However, this gain is at the expense of a decreasing fidelity in resolution. Therefore, to balance the two conflicting requirements, we suggest using a strip that is ‘as thin as the fabrication technology would allow', and to meet the second requirement we propose applying a threshold voltage to enable the signal-processing software to recognize the output voltage.

On the basis of the simulation results we obtained (Fig. 3a–c), we fabricated a dsTENG sensor with the optimized structural dimensions: 25-μm PTFE film, 75-μm Kapton film and 500-μm-wide copper strips. Noting that a higher voltage output is obtained at either larger loads or higher velocities (Fig. 3d), the output performance is measured at varying external loadings and velocities (Fig. 3d–f). When the load becomes sufficiently large, the voltage output converges to *V*_oc_. Further, the maximum output power for varying velocities occurs at varying external loads (Fig. 3e), with a higher power occurring at a higher velocity. The signal frequency is conducted using the fast Fourier transform (see Supplementary Fig. 4) and the result exhibits a strong linear relationship with velocity (Fig. 3f), which is desirable as it indicates that the device will be a reliable velocity sensor.

### Direction measurement and performance

**Figure 4:**
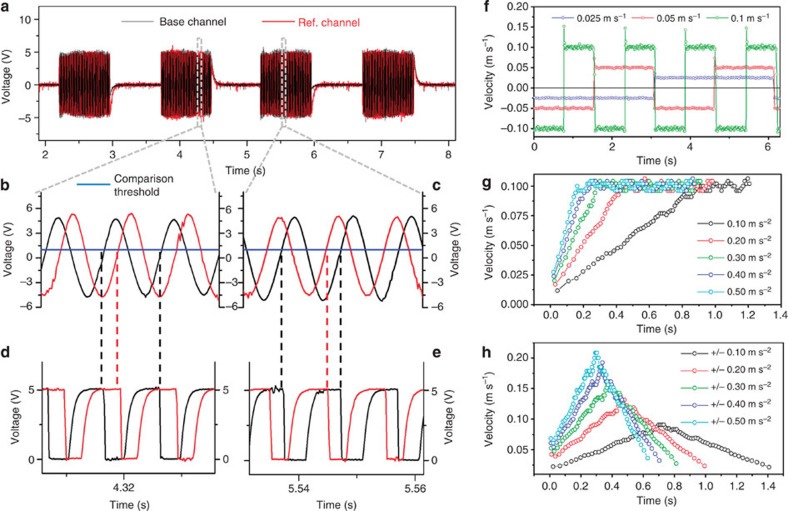
Direction-sensing mechanism and kinematic measured results. (**a**) Two-channel signal measuring from BC and RC. (**b**) Local signal form when the mover is moving ‘forward'. (**c**) Local signal form when the mover is moving ‘backward'. (**d**) Local transformed transistor–transistor logic (TTL) signal when the mover is moving ‘forward'. (**e**) Local transformed TTL signal when the mover is moving ‘backward'. (**f**) Velocity measuring on uniform velocity-reciprocating motion. (**g**) Velocity measurement on uniform acceleration motion. (**h**) Velocity measurement on uniform acceleration and deceleration motions.

To sense motion directions, the device requires both BC and RC to be working simultaneously. The dual channel signal measurement unit (Fig. 4a) records output signals from both channels, as the mover performs reciprocating motion over the stator. Owing to the quarter pitch difference between the two channels, the reference signal occurs either after (Fig. 4b) or before (Fig. 4c) the base signal depending on the motion direction. The signal can be processed by a comparator (Fig. 4d,e and Supplementary Fig. 5), which is an analogue to transistor–transistor logic signal converter on the basis of a given reference voltage. Within one cycle, the motion direction can be uniquely determined by comparing a half-cycle length and the time difference between the ‘falling edges' of the BC and the follow-up RC outputs. The cycles and intervals are automatically timed and processed using the Labview programme for simultaneous magnitude and direction displays of the velocity (Supplementary Fig. 6), with instant velocities computed from the BC pitch width divided by the instant full-cycle time length. The mover is then connected to a programmable linear motor capable of generating varying types of reciprocation that includes uniform velocity motion (Fig. 4f, with error shown in Supplementary Fig. 7), uniform acceleration to a constant velocity motion (Fig. 4g), uniform acceleration to a uniform deceleration motion (Fig. 4h) and so on. The results show a consistently successful tracking of the motion. With the velocity vector fully determined, it is easy to compute the remaining two kinematic parameters of position and acceleration.

Given that the space mismatch of the two channels is 250 μm and the sampling rate is 2,000 Hz, the sensor can sense a directional speed as high as 0.5 m s^−1^. If the direction information is not required, the maximum measurable speed increases to 2 m s^−1^. Further, with the two-channel signal output the velocity sensing is continuous and does not appear to be affected by signal fluctuations from speed changes. This leads to highly reliable velocity measurements. To determine accelerations, we need to record velocities from two full cycles over a 2,000-μm distance, and this constrained the maximum detectable acceleration to 1 m s^−2^. The detection range can be increased by broadening the width of the electrode strip and/or increasing the sampling rate, which is hardware-dependable.

### Reliability

Our test results showed that the technique developed for direction sensing is highly reliable and highly repeatable (Supplementary Fig. 7). Further, with the electrodes for output measurements placed only in the stator, which is protected by the PTFE polymer film, frictional and other abrasive effects are minimized and this ensures a highly reliable and repeatable functioning of the device. We carried out over 86,000 sliding motions under an open-circuit condition without observing measurable output deteriorations (Supplementary Fig. 8). What is seen are some very thin scratches on the copper strips in the motion direction, and this is attributed to uneven assembly of the PTFE layer. The reason the output seems to be largely unaffected is because the copper strips on the mover are for holding (and not conducting) the positive triboelectric charges. Thus, the presence of scratches will not affect the output generation but the charge transfer rate can affect it. As long as the total area of the copper strips remains substantially unchanged, the sensor will work properly. In addition, the electrodes are protected by the PTFE layer to ensure good signal output and the Teflon nanoparticles in the PTFE layer will significantly reduce the friction. The durability performance of our device in comparison with reported TENGs^11^,^30^,^31^,^32^ is listed in Supplementary Table 1.

### Operation demonstration

**Figure 5:**
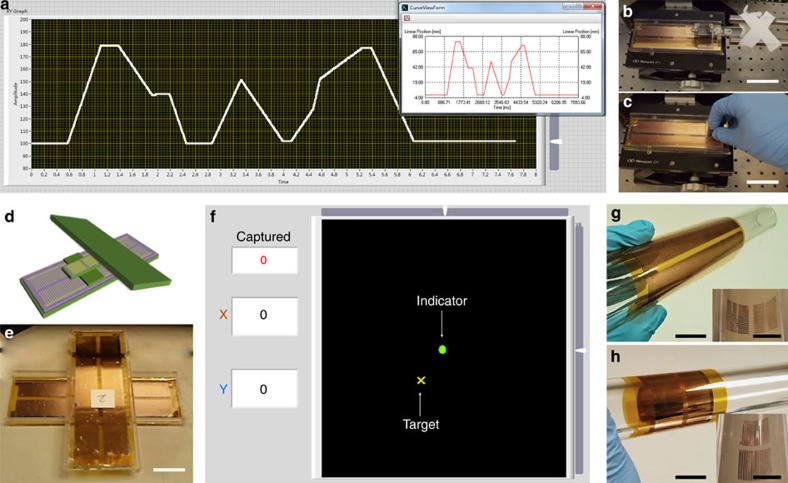
A dsTENG as 1D/2D position sensor. (**a**) 1D position sensor performance as dsTENG was mounted to programmable linear motor. (**b**) dsTENG was mounted to a linear motor (scale bar, 5 cm). (**c**) dsTENG was driven by hand (scale bar, 5 cm). (**d**) Schematic diagram for 2D sensor. (**e**) Experimental set-up for 2D sensor (scale bar, 3 cm). (**f**) User interface for 2D sensor: a capturing game. (**g**) dsTENG can be mounted along the axial of a cylinder (scale bar, 3 cm; inset scale bar, 3 cm). (**h**) dsTENG can be mounted to surround a cylinder (scale bar, 3 cm; inset scale bar, 3 cm).

To demonstrate a working model in real time, we built a 1D position-sensing system on the dsTENG. The sensing system is able to record the mover's position vector (displacement) as driven by a linear motor (Fig. 5a,b, Supplementary Fig. 9 and Supplementary Movie 1) or a random force (Fig. 5c, Supplementary Fig. 10 and Supplementary Movie 2). Figure 5a depicts an accurate consistency between the monitored displacement–time curve and the programmed curve of the linear motor (inset). By integrating two as-fabricated sensors in a vertical connection (Fig. 5d,e), we fabricated a 2D vector sensor that can act as an input terminal for a tracing system. In the demo (Supplementary Movies 3 and 4), the human is allowed to manipulate the indicator either freely or moving towards and catching the target using the 2D sensor (Fig. 5f). The hardware resolution, which is dependent on the width of the strip, is ∼50 dots per inch. It can be further improved via either a hardware (width) redesign or a software approach. In addition, since the dsTENG is fully pliable it can be easily bent to attach to all kinds of surfaces and/or extended in any direction, such as along the axis of a cylinder (Fig. 5g) or its circumference (Fig. 5h).

## Discussion

The existing technologies for the common kinematic sensors are on the basis of electromagnetic induction, capacitor change, resistance change, optical encoding and so on. In contrast, the basic mechanism of our dsTENG unit utilizes the triboelectric effect, which exists between the contact surfaces of two different materials. Currently, not all aspects of our design are competitive to commercial products; however, its unique self-powered feature, flexible micro size and other advantages make our design complementary to and could potentially replace some parts of the traditional kinematic sensor. A comparison of our sensor with commercially available kinematic sensors is tabulated in Supplementary Table 2.

From a structural point of view, commercial products have bulky probes because of the use of magnets, optocouplers or chips for an external power supply. Further, it is not possible to shrink these macro-sized components to the dimensions of the layered structure found in our design, which consists of a three-layered thin film of only a few hundred micrometres thick. It can be fitted into an extremely narrow place such as a bearing to monitor, for example, the displacement. The installation can be as simple as attaching the mover and the stator to the surfaces of two closed moving objects without adversely affecting their separation gaps. In addition, the extremely light mover has minimal effect on the motion of the moving object.

With regard to the signal output, most of commercial sensors are electrically driven and this implies that they need to be connected to an external power source with characteristic feedback signals via the third terminal. As a result, the minimum number of wires attached to the probe of a commercial sensor is three. On the other hand, the triboelectric-based kinematic sensor is able to actively generate characteristic signals directly from motion, which means there is no need to have a wired connection to the mover. Therefore, only two wires are needed to hook it up to the stator for tapping the signal outputs from the electrodes. This advantage reflects not only saving in power, but also simplifies the wire connection for the entire monitoring system.

With regard to the product reliability, the structural wear and tear or the wire fatigue loading during repeated motions is usually the cause of failure in a commercial sensor (unless the sensor is completely sealed and lubricated for long-life operations). Our design contains no wires to the moving part and encounters minimal fatigue loadings during repeated motions. The electrodes for generating signals are well protected by the PTFE films without having to engage the direct frictions. Should they become worn out, only the copper electrodes in the mover require replacement and the process is quite straightforward and economical.

With regard to the issue of cost, a dsTENG kinematic sensor is fabricated from a very small amount of common materials that includes an insulating polymer and a common metal, all in the form of a thin film. The fabrication process is simple and straightforward, and it can be easily scaled up for a large-scale production. This is one of the advantages of our design that few commercial sensors will never be able to beat.

Last but not the least, our thin-film, light-weight kinematic sensor can be easily expanded for 2D motion detection. Further, the bendable films make it easy for attachment to multiple situations such as attaching to a curved surface or for a rotatory-related motion sensing.

Starting from the basic principle of TENG, we develop a fast response demonstration of a self-powered kinematic vector sensor for magnitude and direction tracking and measurements for 1D and 2D motions. Our device is fabricated on highly pliable thin films that can be easily bent to adapt and fit any curved or irregular surfaces. Although the hardware resolution in the dsTENG is ∼50 dots per inch, it can be further improved via a systematic optimization of the structural dimensions of the thin film and copper electrodes. The sensor possesses excellent stability with good repeatability and produces a strong signal durability. We show that it is technologically feasible to sustainably harness triboelectric energy on a macro scale to self-power sensors and small devices for reliable and robust operations.

## Methods

### Fabrication of a dsTENG on flexible films

*Mover*. Cut two columns of hollow strips with 500-μm strip width, 2.5-cm strip length and 1-mm pitch over a piece of 1.5-mm-thick rectangular acrylic sheet (6 cm wide and 20 cm long) using a laser cutter. The two columns are separated with a 5-mm width and are shifted by a ¼ pitch mismatch between each other. Then, put the as-cut mask over a 6-cm width, 20-cm long and 150-μm-thick PTFE film, and then deposit a layer of copper (∼200 nm) using the physical vapour deposition (PVD) method. Remove the mask. Cut the film along its long side to form a 6 cm × 4 cm piece. Mount the film using the back surface.

*Stator*. Cut the mask using the same parameters as in the Mover but without the ¼ pitch mismatch between two-column strips. Then, put the as-cut mask over a 6-cm width, 20-cm long and 75-μm-thick Kapton film and then deposit a layer of copper (∼100 nm) using the PVD method. Carry out the same on the back surface of the Kapton film. This time, shift the mask by a ½ pitch towards the pattern on the front. Remove the mask. Deposit four bus electrodes to individually connect each column strips using mask and PVD. Cover a 6-cm width, 20-cm long and 75-μm-thick PTFE film over the front surface of the as-fabricated Kapton. Mount from the back surface of the Kapton.

### 1D/2D kinematic sensing experimental set-up

*1D motion*. Mount the mover and stator films on the acrylic sheets. Connect each bus electrode with copper wires. Two wires from BC or RC are first connected over resistance, respectively. Then, put the mover over the stator and make sure the strips are all paralleled. Measure the output voltage over the resistance. Connect output wires to the comparator LM393(Texas Instrument comparator) on the non-inverting input pin and common ground of the circuit, with the inverting input pin set as 1 V. Handle the output signal from the LM393 with a data acquisition platform and Labview for display on the screen. Programme the direction reading method (if necessary, reverse the role of the BC and RC). The mover can be driven either by hand or mounted to a driving device such as a linear motor.

*2D motion*. Using two 1D set-up mount them perpendicular to each other.

### Generator output potential simulation methods

‘Comsol Multiphysics 4.2' is induced to simulate and present finite element analysis results for the intersection cases. Charge density of 10 μC m^−2^ was pre-applied on to the surface of the PTFE domains for the triboelectrification charges. The other dimensions including thickness and width are acquired from the real experimental samples.

## Additional information

**How to cite this article:** Jing, Q. *et al.* Self-powered thin-film motion vector sensor. *Nat. Commun.* 6:8031 doi: 10.1038/ncomms9031 (2015).

## Supplementary Material

Supplementary Information Supplementary Figure 1-10, Supplementary Tables 1-2, Supplementary Note 1 and Supplementary References

Supplementary Movie 1 Monitoring positions via a motor-driven motion.

Supplementary Movie 2 Monitoring positions via a randomly-driven motion.

Supplementary Movie 3 Two-dimensional free motion sensing.

Supplementary Movie 4 Two-dimensional position capture via a trace-capture mode.
