# Supplementary material for: Self-powered thin-film motion vector sensor
Source: Nat Commun. 2015 Aug 14;6:8031. doi: 10.1038/ncomms9031 (PMC4557336; doi:10.1038/ncomms9031)
Supplement: Supplementary Information — Supplementary Figure 1-10, Supplementary Tables 1-2, Supplementary Note 1 and Supplementary References [file ncomms9031-s1.pdf]

Supplementary Figures

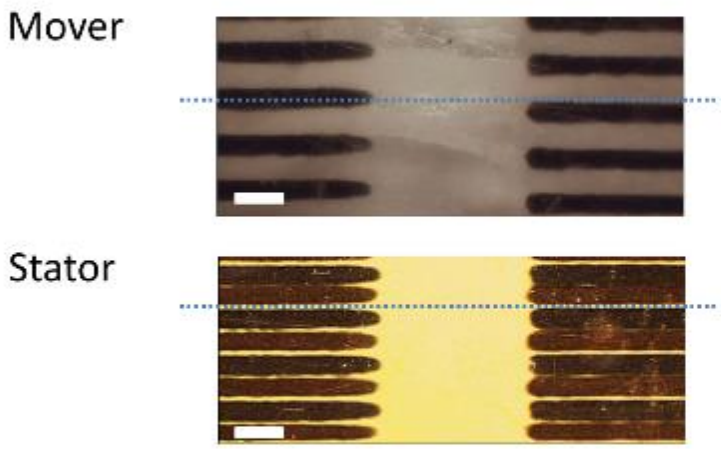

Supplementary Figure 1. Microscopy view of the electrodes on thin films from mover and stator. Electrodes on the mover has a one-fourth pitch shift; Electrodes on the stator are fully aligned. (Scale bar, 1 mm)

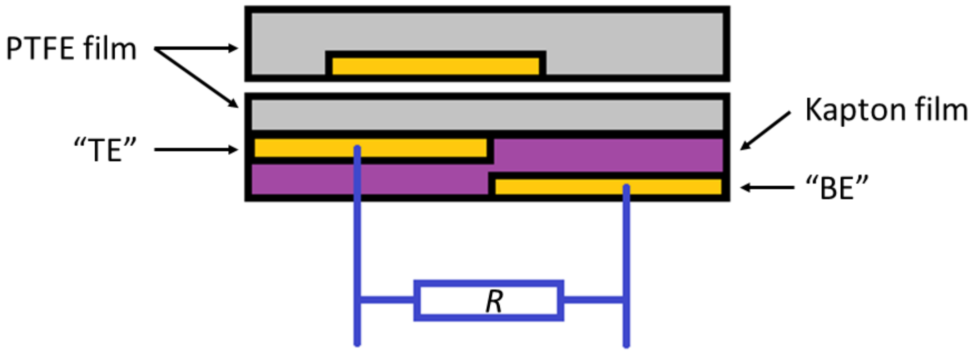

Supplementary Figure 2. Electric connection between top electrode (TE) and bottom electrode (BE). An external load *R* is connected between the TE and the BE.

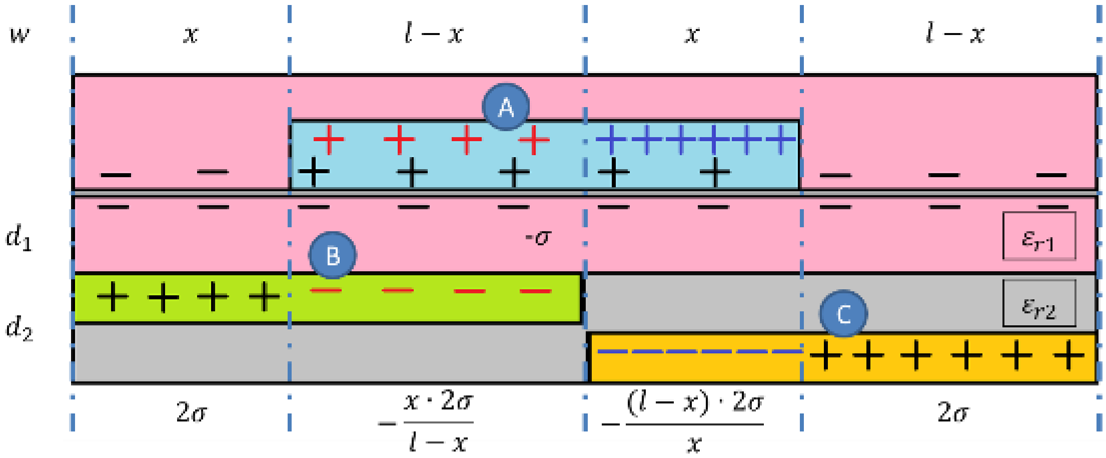

Supplementary Figure 3. Theoretical derivation of output signal differential equations. Detailed derivation is presented in Supplementary Note 1.

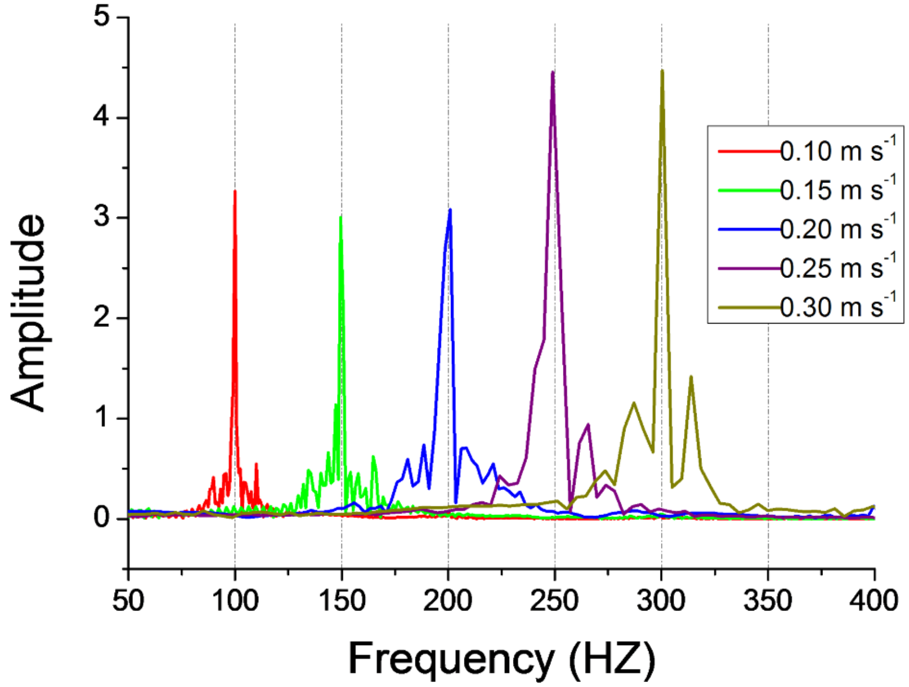

Supplementary Figure 4. FFT toward outputs over varies velocities. The amplitude on y-axis is defined as the spectrum compared with the mean value. Dominant frequencies of these outputs show a great linear relationship with driven velocity.

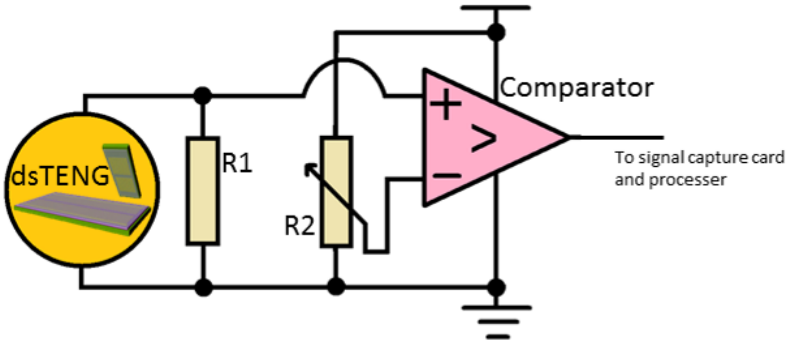

Supplementary Figure 5. Circuit connection between dsTENG channels and comparator. Each of base channel and reference channel has to be connected to an individual comparator.

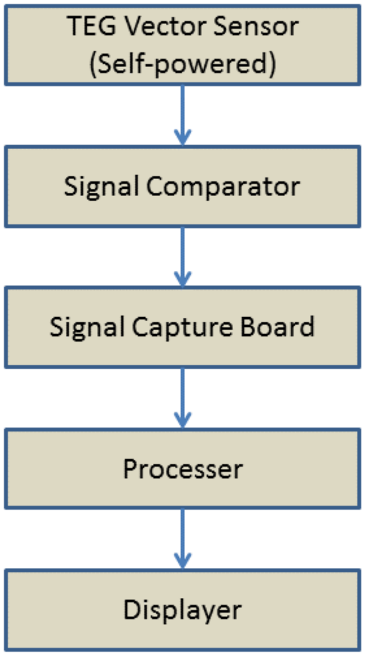

Supplementary Figure 6. Data flow chart for whole signal processing. The signal is proceeded in a consequent approach.

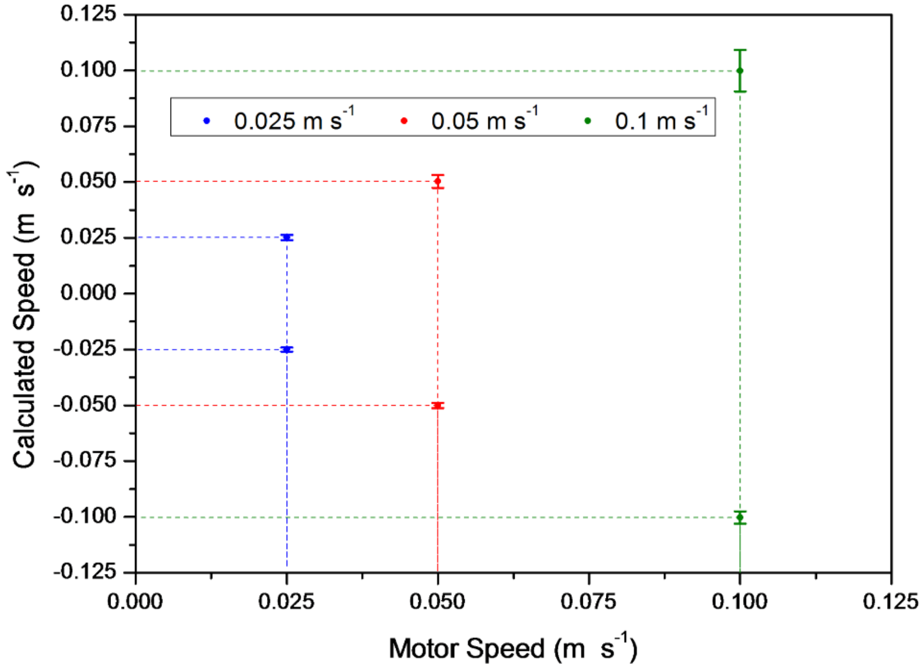

Supplementary Figure 7. Error diagram on repeating measurement of constant velocity. The velocities are measured under several different driven speed. Error bar shows the standard deviation in different tests.

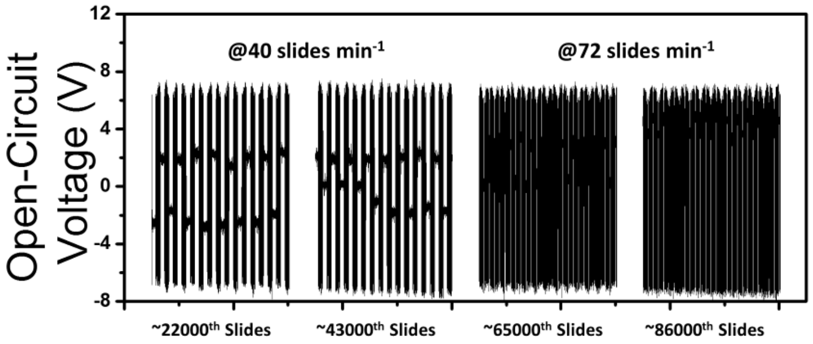

Supplementary Figure 8. Durability test of the device in continuously reciprocating motion and with open-circuit voltage monitored. The first 43000 slides were under 40 slides min^{-1} and the following 43000 slides were under 72 slides min^{-1} compositing a total length of 28 hours.

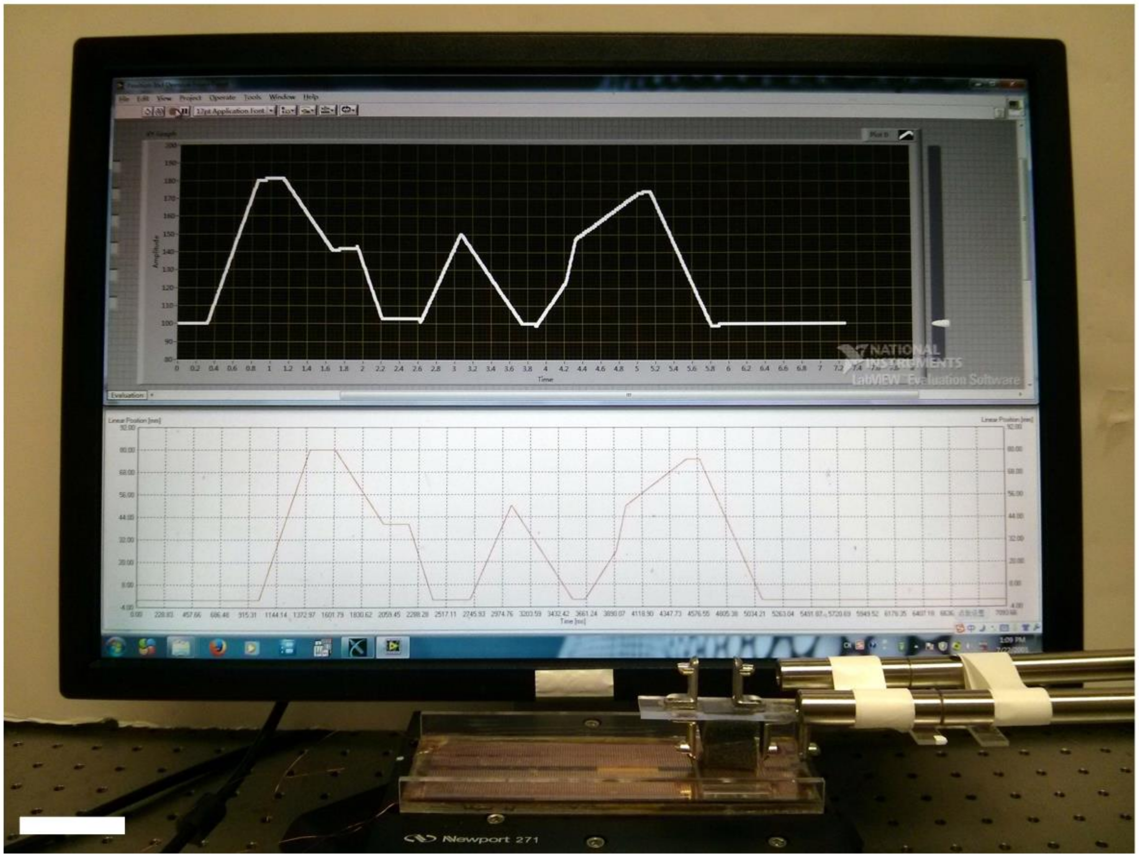

Supplementary Figure 9. dsTENG in recording motion from programed linear motor. Bottom window shows the working curve of the programmable linear motor. Top window shows the real-time measured displacement curve. (Both charts have y-axis as displacement and x-axis as time, scale bar, 5 cm)

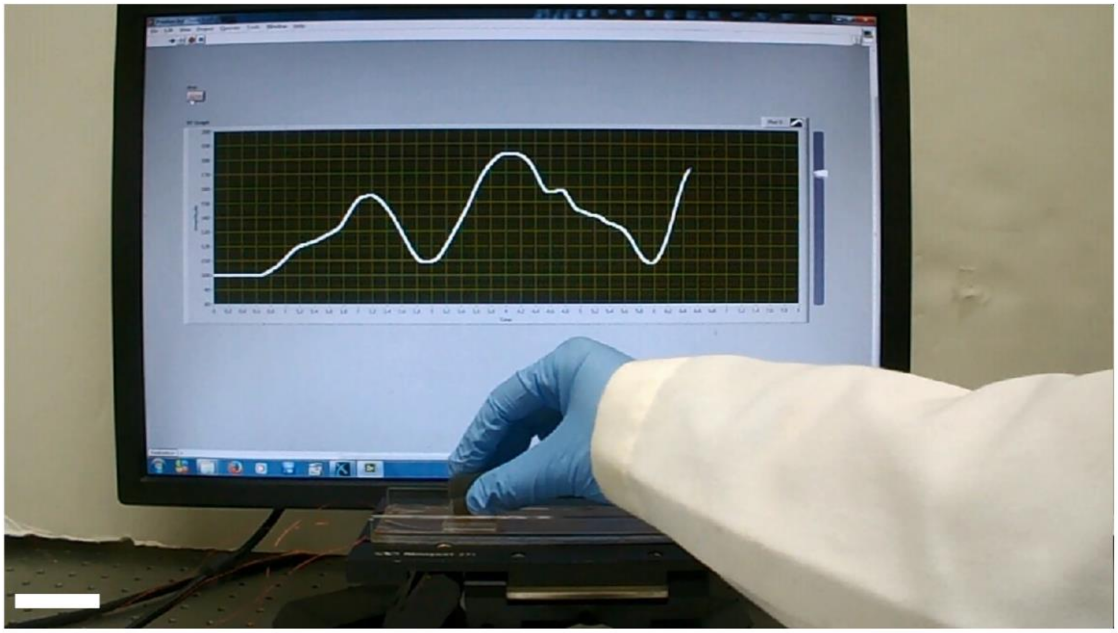

Supplementary Figure 10. dsTENG in recording motion from random driven. Window shows the real-time measured displacement curve. (Chart has y-axis as displacement and x-axis as time, scale bar, 5 cm)

Supplementary Tables

Supplementary Table 1. Comparison of durability performance test results for triboelectric nanogenerators.

System	Mode	Durability Data	Total Signal cycles	Comment
Freestanding Triboelectric Layer-Based Nanogenerators[1]	Sliding	20,000 slides	20,000	Test was conducted with the mover in a gap-contact with the stator.
Cylindrical Rotating TENG[2]	rotating	20,000 rotary cycles	120,000	The TENG generates 6 AC signal cycles per rotary cycle.
Noncontact Free-Rotating Disk TENG[3]	rotating	500,000 rotary cycles	4,000,000	Test was conducted with the mover in a gap-contact with the stator. The TENG generates 8 AC signal cycles per rotary cycle.
Radially Arrayed Rotary TENG[4]	rotating	166,000 rotary cycles	10,000,000	Test was conducted over 10 million AC output cycles at 60 AC signal cycles per rotary cycle.
Thin-Film Motion Vector Sensor (our work)	sliding	86,000 slides	12,900,000	Since 1 slide covers 15 cm distance and generates 150 AC signal cycles, the total number AC signal cycles during the test is approx. 12.9 million.

Remark: As can be seen from the Table, our 86,000-cycle test seems comparable to similar durability tests sourced from published data. Further, if the stability of a “continuously generating AC signals” is considered, our device appears to be among the best performer.

Supplementary Table 2. Characteristic comparison with commercial kinematic sensors

Kinematic Sensor	Power Source	Structural Configuration	Installation	No. of Wired Connections	Wires in Mover
Commercial Sensors	External Power Supply	Bulky and Rigid	Connected to moving objects and require a large space	≥ 3 for velocity sensing<br>≥ 4 for direction sensing	Most have a wire connected mover and constitutes a major source of fatigue failure
Our Sensor	Self-Powered	Thin-Film and Flexible	Can be attached to the gap between the moving objects, such as in a bearing	2 for velocity sensing<br>3 for direction sensing (one common ground for both channels)	No wires are connected to the mover

Supplementary Note

Supplementary Note 1 (Please refer to Supplementary Figure 3)

The output can be theoretically derived by utilizing electrostatic theories. Supplementary Figure 3 shows an instant status of one unit (with length marked as $2l$) of the intersection of TEVS with charge distribution. The copper electrode in the mover iss assigned with mark A, while the two electrodes for output in the stator are marked as B and C. The relative permittivity of PTFE (pink) and Kapton (gray) were set as ε_{r1} and ε_{r2}. A pre-applied charge density on the surface of PTFE between mover and stator is given as $-\sigma$. Relatively, considering the local contact area distribution between copper and PTFE is approximately 1:3, the copper electrode A should carry a total charge amount of $3l\sigma$ in order to balance the negative charge on PTFE. Under open circuit condition, as electrode A has a x mismatch from electrode B, charges redistribute as shown in Supplementary Figure 3. The voltage between A and B, as well as A and C can be described as following,

$$U_A - U_B = \frac{x \cdot 2\sigma \cdot d_1}{(l-x)\varepsilon_0\varepsilon_{r1}} \tag{1}$$

$$U_A - U_C = \frac{(l-x) \cdot 2\sigma \cdot d_1}{x\varepsilon_0\varepsilon_{r1}} + \frac{(l-x) \cdot 2\sigma \cdot d_2}{x\varepsilon_0\varepsilon_{r2}} \tag{2}$$

Where d_1, d_2 are thickness of PTFE film and Kapton film in stator. ε_0 is the permittivity in vacuum. The open circuit voltage output therefore can be calculated as,

$$U_{BC}^0 = U_C - U_B = \frac{x \cdot 2\sigma \cdot d_1}{(l-x)\varepsilon_0\varepsilon_{r1}} - \frac{(l-x) \cdot 2\sigma \cdot d_1}{x\varepsilon_0\varepsilon_{r1}} - \frac{(l-x) \cdot 2\sigma \cdot d_2}{x\varepsilon_0\varepsilon_{r2}} \tag{3}$$

Or

$$U_{BC}^0 = \frac{2\sigma}{\varepsilon_0}\left[\frac{x \cdot d_1}{(l-x)\varepsilon_{r1}} - \frac{(l-x) \cdot d_1}{x\varepsilon_{r1}} - \frac{(l-x) \cdot d_2}{x\varepsilon_{r2}}\right] \tag{4}$$

As there is electronic load between B and C, a general voltage description V^{BC}using capacitor C and charge Q can be raised up using the following equations

$$V^{BC} = -\frac{1}{C_{BC}} \times Q^{BC} + U_{BC}^0 \tag{5}$$

$$V^{BC} = -\frac{1}{\varepsilon_0 w}\left[\frac{d_1}{(l-x)\varepsilon_{r1}} + \frac{d_1}{x\varepsilon_{r1}} + \frac{d_2}{x\varepsilon_{r2}}\right] \times Q^{BC} + \frac{2\sigma}{\varepsilon_0}\left[\frac{x \cdot d_1}{(l-x)\varepsilon_{r1}} - \frac{(l-x) \cdot d_1}{x\varepsilon_{r1}} - \frac{(l-x) \cdot d_2}{x\varepsilon_{r2}}\right] \tag{6}$$

Under open-circuit condition, charge transfer is zero,

$$V_{oc} = \frac{2\sigma}{\varepsilon_0}\left[\frac{x \cdot d_1}{(l-x)\varepsilon_{r1}} - \frac{(l-x) \cdot d_1}{x\varepsilon_{r1}} - \frac{(l-x) \cdot d_2}{x\varepsilon_{r2}}\right] \tag{7}$$

Since

$$C_{BC} = \frac{1}{\frac{1}{C_{BA}} + \frac{1}{C_{CA}}} \tag{8}$$

$$C_{BA} = \frac{\varepsilon_0\varepsilon_{r1}(l-x)w}{d_1} \tag{9}$$

$$C_{CA} = \frac{1}{\frac{d_1}{\varepsilon_0\varepsilon_{r1}xw} + \frac{d_2}{\varepsilon_0\varepsilon_{r2}xw}} \tag{10}$$

$$C_{BC} = \frac{1}{\frac{d_1}{\varepsilon_0 \varepsilon_{r1}(l-x)w} + \frac{d_1}{\varepsilon_0 \varepsilon_{r1} x w} + \frac{d_2}{\varepsilon_0 \varepsilon_{r2} x w}} \tag{11}$$

Combining the results with Ohm's law

$$V^{BC} = I \cdot R \tag{12}$$

And definition of current, the output must satisfy the following equations,

$$I = \frac{\mathrm{d}Q^{BC}}{\mathrm{d}t} \tag{13}$$

$$x = vt \tag{14}$$

$$R \cdot \frac{\mathrm{d}Q^{BC}}{\mathrm{d}t} = -\frac{1}{\varepsilon_0 w}\left[\frac{d_1}{(l-vt)\varepsilon_{r1}} + \frac{d_1}{vt\varepsilon_{r1}} + \frac{d_2}{vt\varepsilon_{r2}}\right] \times Q^{BC} + \frac{2\sigma}{\varepsilon_0}\left[\frac{vt \cdot d_1}{(l-vt)\varepsilon_{r1}} - \frac{(l-vt) \cdot d_1}{vt\varepsilon_{r1}} - \frac{(l-vt) \cdot d_2}{vt\varepsilon_{r2}}\right] \tag{15}$$

Supplementary References

1 Wang, S. H. *et al*. Freestanding triboelectric-layer-based nanogenerators for harvesting energy from a moving object or human motion in contact and non-contact modes. *Adv. Mater.* **26**, 2818-2824 (2014).

2 Bai, P. *et al.* Cylindrical rotating triboelectric nanogenerator. *ACS Nano* **7**, 6361-6366 (2013).

3 Lin, L. *et al*. Noncontact free-rotating disk triboelectric nanogenerator as a sustainable energy harvester and self-powered mechanical sensor. *ACS Appl. Mater. Interfaces* **6**, 3031-3038 (2014).

4 Zhu, G., Chen, J., Zhang, T. J., Jing, Q. S. & Wang, Z. L. Radial-arrayed rotary electrification for high performance triboelectric generator. *Nat. Commun.* **5**, 3426 (2014).
